# Analysis of the effect of serious illness medical insurance on relieving the economic burden of rural residents in China: a case study in Jinzhai County

**DOI:** 10.1186/s12913-020-05675-8

**Published:** 2020-08-28

**Authors:** Yang Li, Guangfeng Duan, Linping Xiong

**Affiliations:** Department of Health Service, Second Military Medical University, Shanghai, 200433 China

**Keywords:** Serious illness medical insurance, Rural resident, Reimbursement effect

## Abstract

**Background:**

In 2003, China established a New Rural Cooperative Medical System (NRCMS) for rural residents to alleviate the burden of medical expenses among rural residents. However, its reimbursement for high medical costs was insufficient. Therefore, China gradually established the Serious Illness Insurance System (SIMIS) based on NRCMS. After receiving payment through NRCMS, patients in rural areas who met the requirements of SIMIS policy would receive a second payment for their high medical expenses. This study aimed to analyze the effect of the implementation of SIMIS on alleviating the economic burden of rural residents in Jinzhai County.

**Methods:**

The study used the inpatient reimbursement data of NRCMS in Jinzhai County, Anhui Province, from 2013 to 2016. We adopted descriptive and regression discontinuity (RD) methods to analyze the payment effect of SIMIS. The RD analysis targeted patients (*n* = 7353) whose annual serious illness expenses were between CNY 10,000 (1414 USD) and CNY 30,000 (4242 USD), whereas the descriptive analysis was used for data of the patients compensated by SIMIS (*n* = 2720).

**Results:**

The results of RD showed that the actual medical insurance payment proportion increased by about 2.5% (lwald = 0.025, *P* < 0.01), inside medical insurance self-payment proportion increased by about 2% (lwald = 0.020, *P* < 0.10), and outside medical insurance self-payment proportion decreased by about 1.6% (lwald = − 0.016, *P* < 0.05). The descriptive results showed that patients with serious illnesses mostly chose to go to a hospital outside the county. The annual average number of hospitalizations was 3.64. The reimbursement mainly came from the NRCMS. The payment amount of SIMIS was relatively small, and the out-of-pocket medical expenses were still high.

**Conclusion:**

The medical technology level of Jinzhai County could not meet the needs of patients with seriously illnesses, the number of beneficiaries of SIMIS was small, and the ability to relieve the burden of medical expenses of the rural residents was insufficient. The high out-of-pocket expenses increased the possibility that only people with good economic conditions could benefit from the reimbursement of SIMIS, resulting in inequity.

## Background

The costs of treatment of some chronic diseases often bring an economic burden to patients [1–3]. The use of medical insurance to reduce the economic burden of patients faced with large medical costs is a problem that every country must consider in the formulation of medical insurance policy. After years of development, China’s medical security system gradually established a New Rural Cooperative Medical System (NRCMS) targeted at rural residents [4]. The establishment of the NRCMS had alleviated the burden of medical expenses of rural residents in China, but the reimbursement for high medical expenses was insufficient [5]. To improve the reimbursement level of rural residents, China subsequently established a Serious Illness Medical Insurance System (SIMIS) based on NRCMS. The new system has two forms of payment: the first one is to pay for the high expense in proportion to the expenses exceeding the specified amount after the payment of NRCMS; the second one is to pay for some diseases in proportion after the payment of NRCMS. In the present study, people who use the SIMIS were collectively referred to as patients with serious illnesses.

Few empirical studies have been conducted on the reimbursement effect of China’s SIMIS, and they have focused on the payment of SIMIS. Analysis of patients’ out-of-pocket costs has been lacking. As such, the present study analyzed the policy effect after the implementation of SIMIS and provided suggestions for policy adjustment. We used the inpatient reimbursement data of NRCMS from 2013 to 2016, after the implementation of SIMIS, provided by Jinzhai medical insurance management center of Anhui Province.

**Fig. 1:**
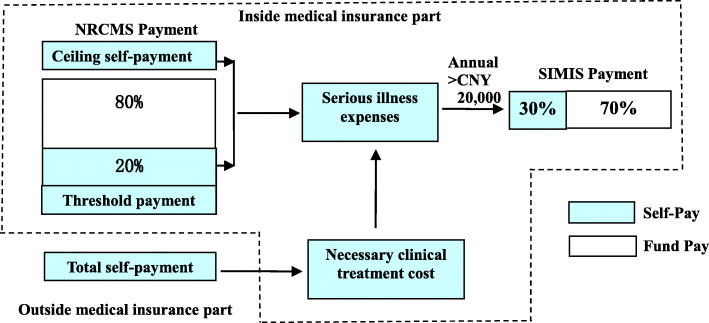
SIMIS payment mode for hospitalization services in Jinzhai

Jinzhai County is located in the west of Anhui Province, China, with a registered population of 683,000 in 2017, including 572,000 in rural areas. About 40,000 people in rural areas live in poverty, half of whom had been driven to poverty by illness [6]. Therefore, China deemed it especially important to establish medical security for rural residents to reduce the poverty incidence. Jinzhai County gradually established a multi-level medical security system composed of NRCMS, SIMIS, and other policies to prevent residents from being driven to poverty owing to illness. Jinzhai County aimed to strengthen the health resources in primary medical institutions to improve medical security [7]. The County’s SIMIS adopted the first form of payment for high expenses, and the payment mode was as follows (Fig. 1):

The total medical expense can be divided into two parts: Inside and outside medical insurance.

### Inside medical insurance

1. NRCMS payment. According to the policy, the total expenses for an inpatient is split into two tiers, namely, the total self-payment and NRCMS payment. NRCMS payment includes the threshold to trigger the NRCMS fund, self-payment under the NRCMS, NRCMS fund payment, and ceiling self-payment.
2. SIMIS payment. The expenses encompassed by the SIMIS are called serious illness expenses. The SIMIS payment comes from self-payment under the NRCMS, ceiling self-payment, and necessary clinical treatment cost in total self-payment. If the annual serious illness expenses of inpatients exceed CNY 20,000 (2828 USD), the SIMIS fund shall be paid in proportion without ceiling limit. If not, the reimbursement scheme falls under the NRCMS payment.

### Outside medical insurance

Total self-payment: The expenses outside the medical insurance are called total self-payment, which the medical insurance cannot reimburse.

## Methods

This study used two methods to analyze the reimbursement effect of SIMIS. First, descriptive analysis was carried out on the inpatient situation of the patients with serious illnesses, describing the different types of inpatient expenses and reimbursement situation in the year, and then analyzing the reimbursement effect. We divided the hospitalization types into five categories: county hospitalization, referring to the inpatients in Jinzhai County; city hospitalization, referring to the inpatients outside Jinzhai County and in Lu’an City; provincial hospitalization, referring to the inpatients outside Lu′an City and in Anhui Province; outside Anhui Province hospitalization, referring to the inpatients outside Anhui Province; and cross regional hospitalization, referring to the cross regional inpatients.

Second, regression discontinuity (RD) [8–10] was used to analyze the relief of medical expenses of rural residents after the implementation of SIMIS. We used RD for the discontinuous characteristics of policy; that is, when the specific index of the research object was greater than the critical value specified by the policy, it would be treated by the policy, and the critical value was the so-called break point (C), or the point at which the line representing X shows a cutoff. As the policy of SIMIS in Jinzhai was to compensate inpatients whose annual serious illness expenses exceeded CNY 20,000 (2828 USD), we set C = CNY 20,000 (2828 USD) as the break point in this study. The participants whose annual serious illness expenses exceeded CNY 20,000 (2828 USD) were included in the experimental group, whereas those whose annual serious illness expenses was less than or equal to CNY 20,000 (2828 USD) were included in the control group. The basic situation of the expenses near the break point was similar. Thus, whether the participants could benefit from the compensation of SIMIS depended on the random allocation of policies, which could be regarded as a quasi-experiment. Owing to the random grouping, the average treatment effect of SIMIS near the break point could be estimated.

We used STATA 15.0 RD for data statistical analysis. The analysis process was as follows:

1. The average and frequency indexes were used to describe the inpatient characteristics and payment status of patients with serious illnesses.
2. We determined the optimal bandwidth (H), which refers to the best distance from the break point. Generally, the smaller the H is, the smaller the deviation of objects (i.e., expenses) on both sides of the break point, but such a scenario may lead to fewer observation objects, resulting in excessive variance. Meanwhile, the larger the H, the smaller the variance, but objects far away from the break point tends to be included, resulting in excessive deviation. Therefore, we used the method proposed by Imbens and Kalyanaraman [11] to select the optimal bandwidth by minimizing the mean square error of two regression functions at the break point. Specifically, in proposing the IK method, Imbens and Kalyanaraman derived the asymptotically optimal bandwidth under squared error loss. This optimal bandwidth depends on unknown functionals of the distribution of the data. They proposed simple and consistent estimators for these functionals to obtain a fully data-driven bandwidth algorithm.
3. We conducted RD analysis. The dependent variables were the actual medical insurance payment proportion (AMIPP), inside medical insurance payment proportion (IMIPP), inside medical insurance self-payment proportion (IMISPP), and outside medical insurance self-payment proportion (OMISPP). The independent variable (grouping variable) was serious illness expenses. The covariates were age, hospital stay, total medical expenses, sex, and inpatient type. Table 1 shows the definition and basic information regarding the variables. In the two intervals (C-H, C) and (C, C + H), the weighted least square method was used for linear regression, with the weight determined by the trigonometric kernel function. The difference between the estimates of dependent variables of the two functions at point C is called the local average treatment effect, also known as “local Wald estimator” (lwald).
4. We conducted the validity test. When conducting RD, we paid attention to the possibility of endogenous grouping. For example, the patients whose serious illness expenses were less than CNY 20,000 (2828 USD) had known the grouping rules in advance, and as such, they could take the initiative to make their serious illness expenses reach CNY 20,000 (2828 USD) and benefit from the compensation policy, resulting in endogenous grouping rather than random grouping of patients near the break point.

**Table 1:** Basic information of variables

Variable	Variable definition	C ≤ 2828 (*n* = 5984)		C > 2828 (*n* = 1370)	
		Mean	SD	Mean	SD
Dependent variable (%)					
AMIPP	Proportion of total medical insurance payment to total medical expenses	48.16	13.52	51.93	11.47
IMIPP	Proportion of total medical insurance payment to total inside medical insurance expenses	59.48	13.19	61.39	11.93
IMISPP	Proportion of inside medical insurance self-payment to total expenses	32.69	11.96	32.81	11.14
OMISPP	Proportion of outside medical insurance self-payment to total expenses	19.15	10.99	15.26	10.71
Independent variable (grouping variable)					
Serious illness expenses	Total of ceiling self-payment, necessary clinical treatment cost, self-payment under NRCMS	1.38	0.27	2.41	0.28
Covariates					
Age	Age of inpatients	49.82	18.39	50.45	17.15
Hospital stay	Length of hospital stay in days	23.26	29.38	26.46	36.34
Total expenses	Total annual inpatient medical expenses	4.78	2.42	7.49	3.27
Sex	Female = 1	0.58	0.49	0.57	0.49
Inpatient type	Inpatient grade (1–5)	4.06	1.06	4.24	1.16

Source: Jinzhai Medical Insurance Management Center

To address the possibility of endogenous grouping, this study used the method proposed by McGrary [12] for testing the discontinuity of the density function of the grouping variable at the break point. The grouping variables were subdivided equidistantly on both sides of break point C. The group distance was B, the center position of each group was noted as variable X_j_, and then the standardization frequency of each group was calculated, which was noted as Y_j_. Using trigonometric kernel and local linear regression on both sides of break point C, the estimated value and standard error of density function could be obtained per the value of the grouped variables. By comparing the estimated values of the density function at the break point, we could determine whether the density function was continuous at the break point.

In addition, even if the conditional density function of covariates at break point C also had a jump, it was not appropriate to attribute all policy effects to the implementation of policies. Indeed, the implicit assumption of RD was that the conditional density of covariates was continuous at the break point. To test this hypothesis, we took each covariate as the dependent variable and the serious illness expenses as the independent variable, and then carried out RD again to check for jumps in its distribution at the break point.

### Sample

The research data came from the medical insurance management center of Jinzhai County, Anhui Province, covering the individual annual hospitalization reimbursement data of NRCMS from 2013 to 2016 (*n* = 73,042 in 2013, *n* = 73,571 in 2014, *n* = 75,330 in 2015, and *n* = 71,928 in 2016). The use of data had been approved by Jinzhai County. Case information included the following: basic information of inpatients, hospitalization, and medical expenses payment. To analyze the SIMIS reimbursement effect, we merged the four-year data (2013–2016, *n* = 29,3871).

We focused our RD on the objects near both sides of the break point. To ensure the same span on both sides of the break point, we selected the patients from the merged data of 2013–2016 whose serious illness expenses was between CNY 10,000 (1414 USD) and CNY 30,000 (4242 USD) as the analysis objects (*n* = 1553 in 2013, *n* = 1797 in 2014, *n* = 2147 in 2015, and *n* = 1856 in 2016, total *n* = 7353). The descriptive analysis of SIMIS reimbursement objects covered the merged data of 2013–2016 (*n* = 468 in 2013, *n* = 535 in 2014, *n* = 831 in 2015, *n* = 886 in 2016, total *n* = 2720). Table 2 shows the distribution of serious illness expenses from 2013 to 2016.

**Table 2:** Distribution of serious illness expenses in Jinzhai County from 2013 to 2016

Expenses	Frequency	Frequency ratio(%)
0.0–	285,501	97.15
1.0–	5983	2.03
2.0–	1370	0.47
3.0–	493	0.17
4.0–	221	0.08
5.0–	303	0.10

Source: Jinzhai Medical Insurance Management Center

## Results

### SIMIS payment

Table 3 presents the descriptive statistics of the patients with serious illnesses in Jinzhai County. These patients were often transferred to other hospitals in different regions. The average number of annual hospitalizations was 3.64. The annual average values for medical expenses for serious illness patients amounted to CNY 96,100 (13,589 USD); for NRCMS payment, CNY 48,000 (6787 USD); for annual average SIMIS payment, CNY 7600 (1075 USD); for inside medical insurance self-payment, CNY 26,200 (3705 USD); and for outside medical insurance self-payment, CNY 14,300 (2022 USD). The average annual expenses of inpatients at the county level was CNY 86,500 (12,231 USD); at the city level, CNY 91,100 (12,882 USD); at the provincial level, CNY 90,700 (12,825 USD); at the level of outside the province, CNY 97,500 (13,787 USD); and at the Cross regional level, CNY 97,300 (13,758 USD). The following proportions were found: that of NRCMS payment to the total medical expenses was 49.95%; that of SIMIS payment to the total medical expenses, 7.90%; that of inside medical insurance self-payment to the total medical expenses, 27.26%; and that of outside medical insurance self-payment to the total medical expenses, 14.88%.

**Table 3:** Annual average inpatient expenses of patients with serious illnesses in Jinzhai County from 2013 to 2016

Items	Total	County	City	Province	Outside province	Cross regional
Hospitalization						
Proportion (%)	100	2.08	5.61	12.23	27.57	52.51
Annual per capita hospital stays	3.64 (2.72)	1.73 (1.65)	2.26 (1.96)	2.12 (1.65)	2.58 (2.41)	4.77 (2.66)
Medical expenses (CNY Ten thousand)						
NRCMS payment	4.80 (3.01)	5.11 (3.01)	5.09 (3.09)	4.83 (2.83)	4.33(3.38)	4.99 (2.79)
SIMIS payment	0.76 (0.11)	0.55 (1.40)	0.60 (0.83)	0.57 (0.63)	0.87 (1.50)	0.76 (0.96)
Inside medical insurance self-payment	2.62 (1.17)	2.33 (1.16)	2.35 (0.90)	2.36 (0.72)	2.78 (1.28)	2.61 (1.01)
Outside medical insurance self-payment	1.43 (1.37)	0.66 (0.57)	1.07 (0.42)	1.31 (1.19)	1.77 (2.36)	1.37 (1.98)
Total	9.61 (5.77)	8.65 (4.58)	9.11 (4.67)	9.07 (4.62)	9.75 (7.59)	9.73 (4.97)
Payment proportion (%)						
NRCMS payment	49.95 (10.46)	59.08 (16.25)	55.87 (9.09)	53.25 (9.37)	44.41 (10.86)	51.28 (9.21)
SIMIS payment	7.90 (5.49)	6.36 (5.00)	6.59 (5.20)	6.28 (4.80)	8.92 (5.93)	7.81 (5.32)
Inside medical insurance self-payment	27.26 (10.85)	26.94 (18.49)	25.80 (9.35)	26.02 (9.95)	28.51 (11.64)	26.82 (10.36)
Outside medical insurance Self-payment	14.88 (11.06)	7.63 (7.76)	11.75 (9.22)	14.44 (10.76)	18.15 (11.50)	14.08 (10.92)

Source: Jinzhai Medical Insurance Management Center

Note:1 USD = CNY 7.077.The values in brackets are SDs

### Analysis of RD

#### SIMIS effect

**Fig. 2:**
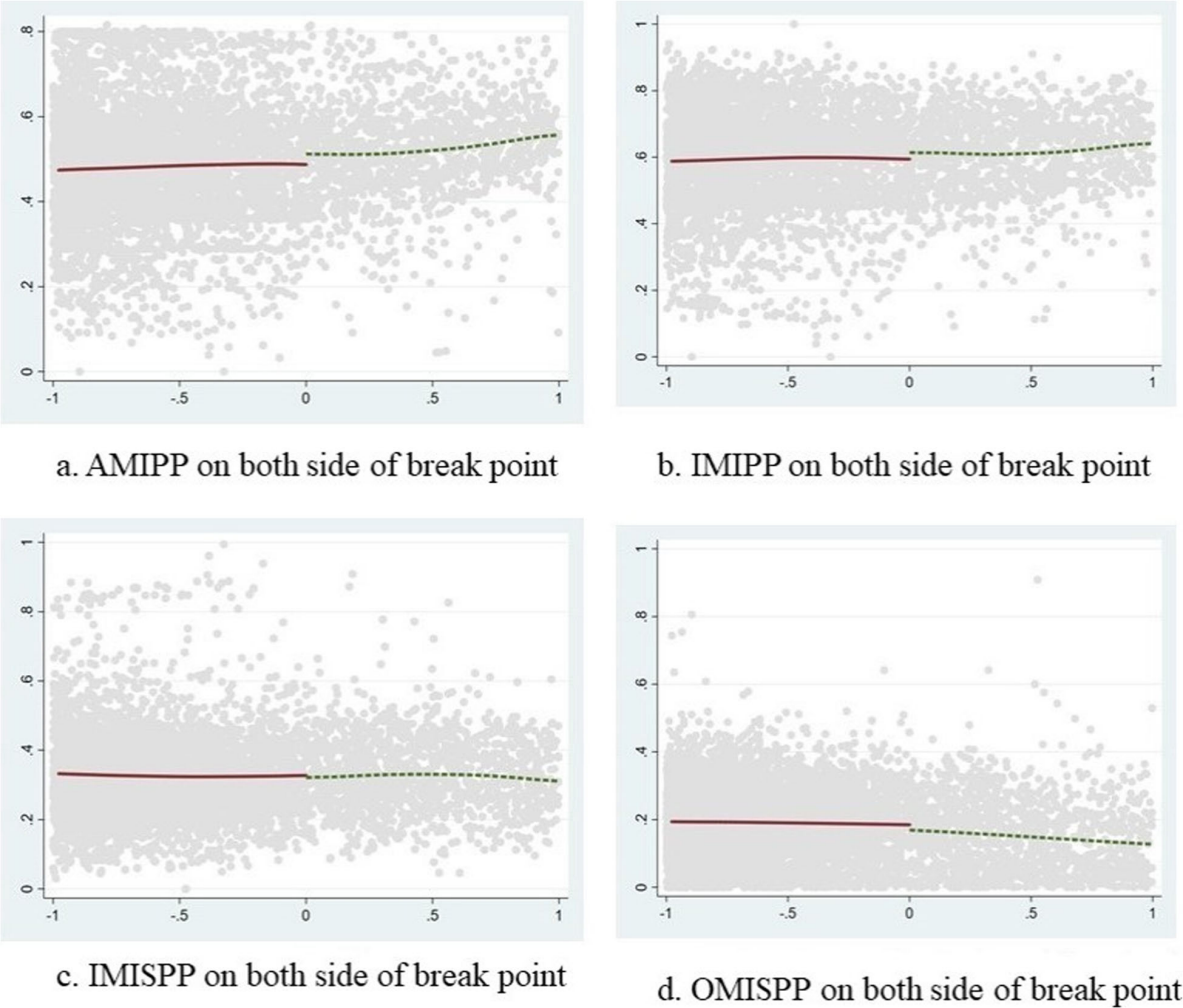
Values of (**a**) Actual Medical Insurance Payment Proportion (AMIPP), **b** Inside Medical Insurance Payment Proportion (IMIPP), **c** Inside Medical Insurance Self-Payment Proportion (IMISPP), and **d** Outside medical insurance Self-Payment proportion (OMISPP) on both sides of the break point

Table 4 gives the estimated effect of policy treatment. In the absence of covariates, the lwald estimate of the AMIPP at the break point was 0.025, which was significant at the level of 1%. As shown in Fig. 2a, the reimbursement on the right side of the break point was slightly higher than that on the left side, indicating that the implementation of SIMIS improved the actual payment level, at about 2.5%. The lwald estimate of IMIPP was 0.020. As shown in Fig. 2b, the reimbursement on the right side of the break point was slightly higher than that on the left side, indicating that the implementation of SIMIS had improved the actual payment level to some extent, at about 2%, but remained slightly lower than the actual payment level of medical insurance. The lwald estimate of IMISPP was − 0.006, and it was not significant. As shown in Fig. 2c, the level of self-payment inside medical insurance was almost the same on the left and right sides of the break point, indicating that the implementation of SIMIS had little impact on the level of self-payment inside medical insurance. The lwald estimate of OMISPP was − 0.016, which was significant at the level of 5%. As shown in Fig. 2d, the level of self-payment outside medical insurance on the right side of the break point was slightly lower than that on the left side, indicating that the implementation of SIMIS reduced the level of self-payment outside medical insurance to a certain extent, at about 1.6%.

**Table 4:** RD results of SIMIS

lwald	AMIPP (H = 0.6581)	IMIPP (H = 0.5345)	IMSPP (H = 0.5949)	OMISPP (H = 1.079)
No covariates added	0.025*** (0.010)	0.020* (0.011)	−0.006 (0.009)	−0.016** (0.006)
Covariates added	0.022*** (0.008)	0.014** (0.006)	− 0.006 (0.009)	− 0.011** (0.005)

Note: **** represents significance at the level of 1%, ** represents significance at the level of 5%, and * represents significance at the level of 10%. H represents the optimal bandwidth. The values in brackets are SDs of lwald

### Validity test

#### Independent variable continuity test

**Fig. 3:**
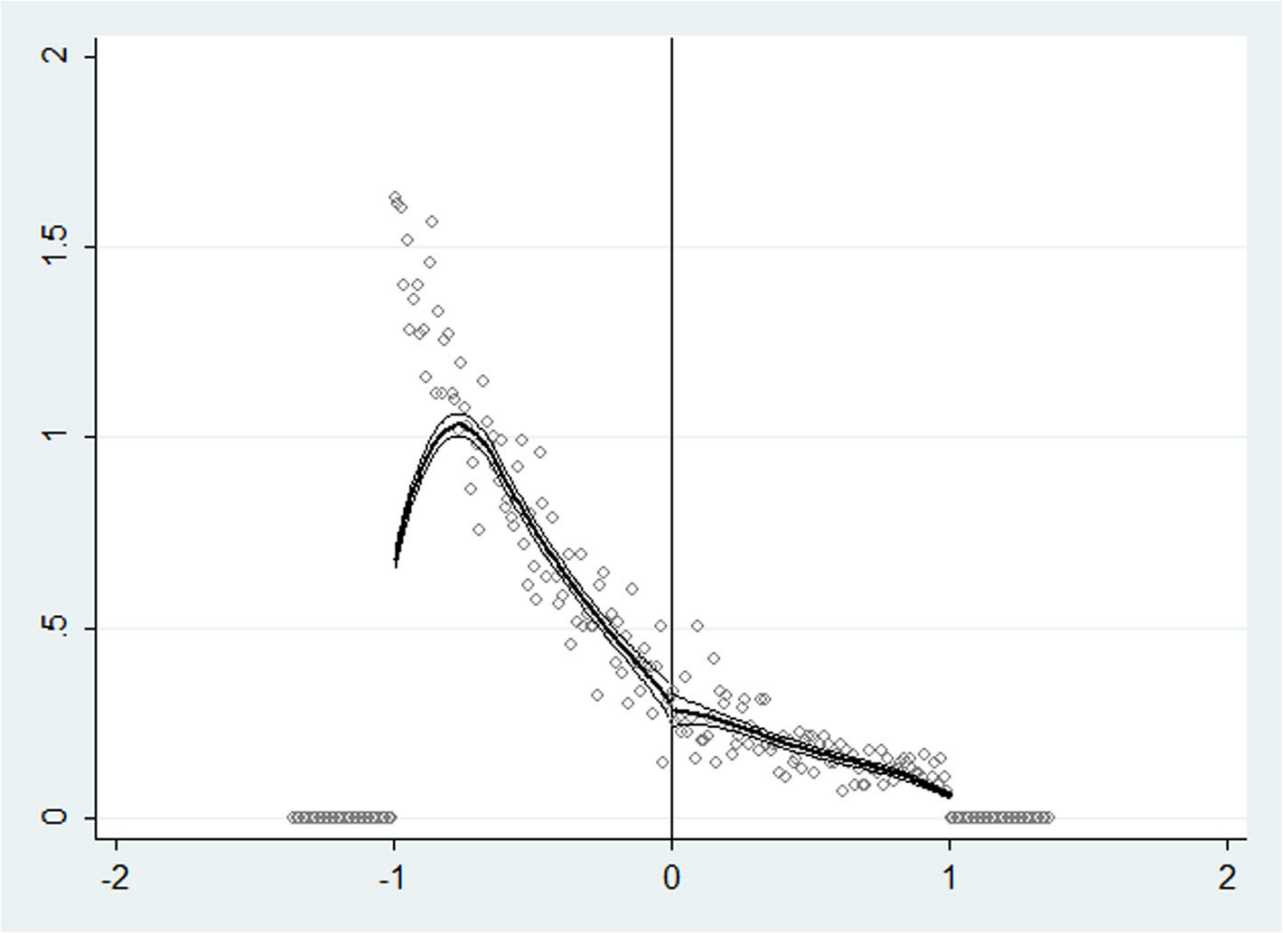
McGrary test of independent variables

We used the method of McGrary (2008) to test whether the probability density function of the independent variables was continuous at the break point. The results showed $\hat{\theta}$= − 0.054, and the standard error was 0.11. Thus, the assumption of continuity of the independent variable density function at the break point could be accepted. As shown in Fig. 3, the confidence intervals of the estimated values of the independent variable probability density functions on both sides of the break point mostly overlapped, indicating that the patients were randomly assigned on both sides of the break point, and there was no endogenous grouping.

#### Covariate continuity test

As shown in Table 5, the estimated lwald was not significant for age, sex, hospital stay, total expenses, and inpatient type. Thus, SIMIS had no impact on the covariates, and the policy effect could be attributed to the implementation of SIMIS.

**Table 5:** Covariate continuity test results

lwald	AMIPP H = 0.6581	IMIPP H = 0.5345	IMSPP H = 0.5949	OMISPP H = 1.079
Age	1.690 (14.516)	3.008 (16.197)	2.355 (15.300)	−2.93 (11.38)
Total expenses	606.16 (1971.54)	1209.01 (2135.78)	968.03 (2045.23)	−907.36 (1581.57.)
Hospital stay	0.943 (2.897)	2.082 (3.241)	1.433 (3.058)	2.176 (2.225)
Inpatient type	−0.041 (0.083)	−0.033 (0.094)	− 0.037 (0.089)	−0.052 (0.066)
Sex	0.035 (0.039)	0.031 (0.043)	0.0358 (0.041)	0.035 (0.030)

Note: The dependent variables are age, total expenses, hospital stay, inpatient type, and sex. The independent variables are serious illness expenses. Values outside the bracket are the estimated values of lwald, and those inside the bracket are the estimated values of standard error

## Discussion

The RD results showed that the SIMIS improved both AMIPP and IMIPP. The treatment effect values (lwald) were 0.025 and 0.020, respectively, and the proportion of payments increased by only 2.5 and 2%. Moreover, the gap between the two sides of the break point (Fig. 2a and b) was small after the implementation of SIMIS. Although the SIMIS reduced the actual out-of-pocket expenses of patients, the descriptive results showed that the level of reimbursement in the case of patients with serious illnesses was higher from the NRCMS. The average annual NRCMS payment was CNY 48,000 (6787 USD), and that of the SIMIS payment was CNY 7600 (1075 USD). The SIMIS reimbursement accounted for about 14% of the total reimbursement. The average annual actual out-of-pocket expenses amounted to CNY 40,500 (5727 USD). In 2016, the average annual income of rural families in Anhui Province was about CNY 32,000 (4525 USD) [13], which meant that the annual income of patients with serious illnesses was lower than the actual self-paid medical expenses. Moreover, the SIMIS could not help prevent the catastrophic health expenditure of the people. Similar to Jinzhai’s SIMIS, the average annual NRCMS payment in Beijing is CNY 42,808 (6053 USD), the SIMIS payment is CNY 5212 (737 USD), and average annual actual out-of-pocket expenses is about CNY 41,372 (5850 USD) [14]. Thus, although the SIMIS reduced the economic burden of patients to a certain extent, it had no significant effect on relieving catastrophic health expenditure [14]. Meanwhile, Shanghai’s SIMIS pays according to disease treatment, including dialysis treatment of severe uremia, anti-rejection treatment of kidney transplantation, treatment of malignant tumor, and treatment of some mental diseases. The per capita reimbursement of SIMIS in Shanghai is reportedly CNY 453 (64 USD), but the reimbursement of NRCMS in Shanghai is relatively high [15].

Outside China, the Ayushman Bharat-Pradhan Mantri Jan Arogaya Yojana (PMJAY) was launched by the central government of India in 2018 as a national Publicly Funded Health Insurance (PFHI) Scheme. The stated objectives of PMJAY are to reduce the financial burden on poor and vulnerable groups for access to quality health services. However, the out-of-pocket expenditure and incidence of catastrophic health expenditure did not decrease with enrollment under PMJAY or other PFHI Schemes [16]. In the US, the Affordable Care Act (ACA) sought to protect families from high and rising health care expenditures by expanding health insurance coverage through the Medicaid program and reducing underinsurance through various avenues. Among low-income patients, Marketplace implementation is associated with 601 USD lower out-of-pocket spending [2]. The ACA’s insurance Marketplaces have also been associated with improved financial protection among low-income surgical patients eligible for both cost-sharing and premium subsides, but not in middle-income patients eligible for only premium subsides [2].

We also found that SIMIS reduced OMISPR at the break point, and the treatment effect value was − 0.0016, with self-payment decreasing by about 1.6%. As shown in Fig. 2d, the gap between the two sides of the break point was very small, indicating that although the coverage of the SIMIS had been expanded compared with that of the NRCMS, the range was small. The per capita outside medical insurance for patients with serious illnesses was about CNY 14,300 (2022 USD), which was relatively high. Moreover, our results revealed that the inpatients who could benefit from the compensation of SIMIS accounted for about 0.93% of the total inpatient population.

Regarding hospitalization, 45.41% of the patients with serious illnesses in rural areas directly chose to be hospitalized outside Jinzhai County, whereas 52.51% chose to be transferred to hospitals outside the county. These results could be explained by, first, the medical treatment capacity in Jinzhai County being unable to meet the medical needs of patients with seriously illnesses, and second, the large number of migrant workers in Jinzhai County. Meanwhile, we also found that the annual per capita hospital stays of patients with serious illnesses was 3.64, and the annual per capita hospital stays across regions was 4.77. The results may be related to the fact that most of the patients with serious illnesses were chronically and critically ill.

In addition, the NRCMS payment was found to be related to the type of hospitalization. This result may be linked to the policy orientation of medical insurance; that is, the reimbursement for hospitalization in different places is lower than that of hospitalization in local areas to guide patients to seek medical treatment in local areas. Nonetheless, patients with serious illnesses chose to be hospitalized outside the county, which showed that the guidance of NRCMS had little impact on patients with serious illnesses. Thus, these patients tend to pay more attention to the level of medical technology rather than their economic situation. Research has also shown that most rural patients who choose to see a doctor have better economic condition, so they tend to pay more attention to the level of hospital medical technology [17, 18]. In Jinzhai County, 22% of rural residents work across different provinces all the year round [19]. The economic condition of migrant workers has been reported as relatively good, which may also lead to more migrant workers choosing to go to different places for medical treatment [20]. Therefore, people with better economic conditions were more likely to benefit from the SIMIS, whereas patients with poor economic conditions may choose not to see a doctor, leading to inequity [21, 22].

In analyzing the payment effect of SIMIS, the study had a number of limitations. RD only used part of the data on both sides of the break point to analyze the effect of SIMIS on relieving rural residents’ economic burden. As such, the analysis only reflected the policy effect near the break point. In addition, some of people with serious illnesses and living in poverty, as determined by the government, who have expenses exceeding CNY 5000 (707 USD) could avail of the SIMIS payment. This condition may affect the results of RD analysis, although the number these people in poverty is extremely low, and their impact on the results would be limited.

## Conclusion

As a supplemental policy arrangement to alleviate the economic burden of patients with high medical expenses, the SIMIS has increased the reimbursement amount for some patients with high medical expenses. However, the local medical technology level of Jinzhai County cannot meet the needs of medical services for patients with serious illnesses. Moreover, the range of beneficiaries of SIMIS was small, and its ability to alleviate the economic burden of rural residents was limited. The high out-of-pocket medical expenses increased the possibility that only people with better economic conditions can avail of the reimbursement of SIMIS, resulting in inequity. Therefore, the policy of SIMIS should be modified with consideration for reducing the threshold, expanding the scope, improving the accuracy, and promoting the fairness of payments.

## Data Availability

The data that support the findings of this study are available from Health Committee of Jinzhai County but restrictions apply to the availability of these data, which were used under license for the current study, and so are not publicly available. Data are however available from the authors upon reasonable request and with permission of Health Committee of Jinzhai County.

## Abbreviations

AMIPP: Actual Medical Insurance Payment Proportion
IMIPP: Inside Medical Insurance Payment Proportion
IMISPP: Inside Medical Insurance Self-Payment Proportion
NRCMS: New Rural Cooperative Medical System
OMISPP: Outside medical insurance Self-Payment proportion
RD: Regression Discontinuity
SIMIS: Serious Illness Medical Insurance System
